# RNA-Binding Protein Motifs Predict microRNA Secretion and Cellular Retention in Hypothalamic and Other Cell Types

**DOI:** 10.3390/biomedicines12040857

**Published:** 2024-04-12

**Authors:** Wenyuan He, Denise D. Belsham

**Affiliations:** 1 Department of Physiology, University of Toronto, Toronto, ON M5S 1A8, Canada; wenyuan.he@mail.utoronto.ca; 2 Department of Medicine, University of Toronto, Toronto, ON M5S 1A8, Canada

**Keywords:** microRNA, hypothalamus, RNA-binding protein, motif, secretion, retention

## Abstract

Cellular microRNAs (miRNAs) can be selectively secreted or retained, adding another layer to their critical role in regulating human health and disease. To date, select RNA-binding proteins (RBPs) have been proposed to be a mechanism underlying miRNA localization, but the overall relevance of RBPs in systematic miRNA sorting remains unclear. This study profiles intracellular and small extracellular vesicles’ (sEVs) miRNAs in NPY-expressing hypothalamic neurons. These findings were corroborated by the publicly available sEV and intracellular miRNA profiles of white and brown adipocytes, endothelium, liver, and muscle from various databases. Using experimentally determined binding motifs of 93 RBPs, our enrichment analysis revealed that sEV-originating miRNAs contained significantly different RBP motifs than those of intracellularly retained miRNAs. Multiple RBP motifs were shared across cell types; for instance, RBM4 and SAMD4 are significantly enriched in neurons, hepatocytes, skeletal muscle, and endothelial cells. Homologs of both proteins physically interact with Argonaute1/2 proteins, suggesting that they play a role in miRNA sorting. Machine learning modelling also demonstrates that significantly enriched RBP motifs could predict cell-specific preferential miRNA sorting. Non-optimized machine learning modeling of the motifs using Random Forest and Naive Bayes in all cell types except WAT achieved an area under the receiver operating characteristic (ROC) curve of 0.77–0.84, indicating a high predictive accuracy. Given that the RBP motifs have a significant predictive power, these results underscore the critical role that RBPs play in miRNA sorting within mammalian cells and reinforce the importance of miRNA sequencing in preferential localization. For the future development of small RNA therapeutics, considering these RBP-RNA interactions could be crucial to maximize delivery effectiveness and minimize off-target effects.

## 1. Introduction

MicroRNAs (miRNAs) are small, non-coding RNAs that regulate gene expression. Although miRNAs are produced intracellularly and act on messenger RNAs (mRNAs) to regulate protein levels, they can also be secreted in small extracellular vesicles (sEVs). These sEVs play a crucial role in cell-to-cell communication by transporting bioactive cargoes from donor to recipient cells [1]. MiRNAs, along with lipids and other RNA species, are among the signals conveyed through these sEVs [2,3]. The miRNA content of sEVs is known to be modulated by physiological signals, such as exercise, and pathologies, like cancer [2,4,5]. The mechanisms underlying miRNA loading into exosomes remain unclear, despite several hypotheses regarding RNA sorting mechanisms, such as RNA-binding proteins (RBPs), KRAS status, and neutral sphingomyelinase 2 [2,3,6,7]. RBPs constitute a notable group of proteins capable of regulating specific miRNA localization. For example, HnRNPA2B1 can bind to particular miRNA sequences, also allowing the protein to attach to ceramide-enriched membrane regions, thereby directing miRNAs towards exosomal packaging [8]. To date, other RNA-binding proteins, including SYNCRIP, YBX-1, and MVP, have been independently demonstrated to regulate the secretion of specific miRNAs [9,10,11]. However, it is unclear to what extent RBPs contribute to the overall sorting of miRNAs between the intracellular and extracellular compartments. Furthermore, the regulation of miRNA secretion within neuronal models has yet to be explored [12]. Neurons are particularly compelling targets given that the brain stands out as one of the few tissues with a significant baseline presence of high molecular weight Argonaute complexes, indicating enhanced miRNA activity, and neurons have a higher miRNA turnover rate [13,14].

In this study, preferential miRNA secretion and cellular retention were studied in mHypoE-46 neurons, a well-characterized model of NPY/AgRP hypothalamic neurons important for regulating energy homeostasis [15,16,17]. The exosome content of these neurons has been assessed and was used to analyze exosome signaling between NPY and POMC neurons [18]. Additional sEV and intracellular miRNA profiles were obtained from Garcia-Martin et al., 2021, for white and brown adipocytes and endothelium, liver, and muscle cells. Using the experimentally determined binding motifs of 93 RBPs, enrichment analysis revealed that sEV-enriched miRNAs contained significantly different RBP motifs than those found in intracellularly enriched miRNAs [19]. Machine learning modeling of the RBP motifs, using Random Forest and Naive Bayes was used to assess predictive power of these RBP motifs in predict preferential miRNA localization, scoring the area under the ROC curve and produced values between 0.77 and 0.84, which indicates a high predictive accuracy.

## 2. Methods

### 2.1. Cell Culture and miRNA Microarray

The generation and characterization of the mHypoE-46 neurons were previously described. In brief, this model expresses NPY, AgRP, and Tbx3, a marker of the arcuate nucleus (ARC) [15,16,17]. mHypoE-46 neurons (RRID:CVCL_D459) were cultured on fresh Dulbecco’s Modified Eagle Medium (DMEM) (MilliporeSigma, Oakville, ON, Canada) supplemented with 5% fetal bovine serum (FBS) (Gibco, Burlington, ON, Canada) for 24 h, and the cell content was measured. Neurons were incubated at 37 °C in 5% CO_2_. A total of 100 ng of the provided RNA was used for the GeneChip miRNA Array 4.0, conducted by the Centre for Applied Genomics at the Hospital for Sick Children in Toronto, ON, Canada. The data were analyzed using the Transcriptome Analysis Console (TAC) version 4.0.3, employing RMA + DABG analysis, focusing on mouse data only. The resulting miRNA profile was used for downstream analysis. The sEV miRNA profile for mHypoE-46 neurons has previously been published [18]. In summary, mHypoE-46 neuronal cells were cultured to 70–80% confluency on 100 mm culture dishes (Sarstedt, Montreal, QC, Canada). Before treatment, the cells were washed with pre-warmed 1x phosphate-buffered saline. The cells were then incubated in DMEM containing 5.5 mM glucose and supplemented with 5% exosome depleted FBS (Gibco) for 24 h. At the time of collection, the conditioned media were collected and centrifuged at 1000× *g* for 10 min to remove cells, then filtered through a 0.2 μm syringe filter to eliminate debris. sEVs from the media were isolated using the Cell Culture Media Exosome Purification Midi Kit (Norgen Biotek, Thorold, ON, Canada) [18]. The concentration and the size of the particles have previously been characterized [18]. 

### 2.2. Classification of Preferentially Secreted and Cellular miRNAs

Although the same amount of total RNA was used for quantification, the total signal produced by sEV-derived miRNA was significantly lower than that produced by the intracellular miRNAs. Therefore, a direct comparison of absolute miRNA levels might bias the results towards an apparent enrichment of miRNAs within cells. Consequently, our analysis chooses to compare the percentage composition of each miRNA, similar to the read-per-million approaches employed in RNA sequencing. Three replicates for each profile were used in this analysis. Only miRNAs that were reliably detected above background (DABG) values (*p* < 0.05) were selected to determine the total signals present in each profile. The relative composition of an miRNA is determined by dividing the signal of that miRNA by the total reliable signals. If an miRNA was present in the cell but not in the sEV, its sEV composition is recorded as 0%. The percentage composition of each miRNA was then statistically compared using unpaired, two-tailed, *t*-test assuming the same standard deviation, before adjusting for multiple comparisons using the false discovery rate approach, a two-stage setup method by Benjamini, Krieger, and Yekutieli (*q* < 0.05). miRNA profiles from additional cell types were obtained from Garcia-Martin et al., adhering to the pre-existing statistical analysis and grouping methods for independent validation. These profiles include differentiated 3T3-L1 cells (white adipocytes), immortalized differentiated brown adipocytes, differentiated C2C12 cells (skeletal muscle), SVEC (endothelial cells), and AML12 cells (hepatocytes) [19]. Garcia et al. applied an arbitrary fold change and *p*-value cut-off to classify preferentially secreted/intracellularly retained miRNAs based on qPCR results [19]. Not all annotated miRNAs in the mHypoE-46 neurons have been experimentally validated, and some targets may be small non-coding RNAs [20].

### 2.3. Pathway Enrichment Analysis for miRNAs

The Kyoto Encyclopedia of Genes and Genomes (KEGG) functional pathways were analyzed using DIANA-miRPath v4.0 [21]. This analysis incorporated data from four independent databases: miRTarBase 2022 [22], Tarbase v8.0 [23], microT-CDS 2023 [24], and TargetScan v8.0 [25]. While miRTarBase and Tarbase provide experimentally validated miRNA-target interactions, microT-CDS and TargetScan offer predictions of potential targets. miRNA targets from each database were used for an independent KEGG analysis. Threshold settings varied across the databases: miRTarBase included all targets; Tarbase focused on direct targets; microT-CDS applied a threshold of 0.95; and TargetScan considered only conserved targets. A false discovery rate was employed to adjust for multiple testing, with *q* < 0.05 deemed statistically significant. Pathways that were significantly enriched across different algorithms were compiled to identify common pathways across multiple databases.

### 2.4. Motif Discovery

The MEME suite and the accompanying Simple Enrichment Analysis (SEA) were used to determine RBP motifs associated with intracellular-enriched miRNA, accessible via the MEME suite web tool (v5.5.5, https://meme-suite.org/meme/tools/sea, accessed between January–March 2024) [26]. When examining sEV-enriched miRNAs, the intracellularly enriched miRNAs were used as the background sequences, and vice versa. The neutral class was excluded from the background to avoid the potential misclassification of preferentially localized miRNAs as neutral. Additionally, shuffled sequences were not considered for the background, acknowledging that miRNA sequences do not occur in random AUCG patterns. The analysis used the CISBP-RNA database for a single species, *Mus musculus*, and 10% of the data from each group was reserved as a holdout group [27]. The E-value was chosen as a correction measure for the number of motifs evaluated, offering a more stringent assessment than the false discovery rate in this scenario. Datasets from Garcia-Martin et al., 2021, underwent the same SEA process using the MEME suite, analogous to the procedure applied to the mHypoE-46 neuron datasets [19].

### 2.5. Machine Learning Modeling

The WEKA 3.8.6 package was used for the establishment and evaluation of machine learning models [28]. Data were formatted accordingly in Python with the aid of ChatGPT 4.0. Each statistically enriched RBP, both intracellular and sEV-enriched, was used as a feature. The strength of the RBP motif, previously determined in the simple enrichment analysis, is represented as a decimal. Any value below the statistical threshold is tagged as 0. Default settings for WEKA were employed for the modeling. Random Forest and Naïve Bayes were the two algorithms formally evaluated, though other models generally showed similar performances. To gauge the effectiveness of these models, ten-fold cross-validation was used, a technique that divides the dataset into ten parts, using each in turn for validation while training on the remaining nine. While further fine tuning might improve the predictive accuracy of the presented model, the authors believe the current iteration accurately represents the true predictive power of RBPs.

## 3. Results

### 3.1. Preferential Retention and Secretion of Neuronal miRNAs

**Figure 1:**
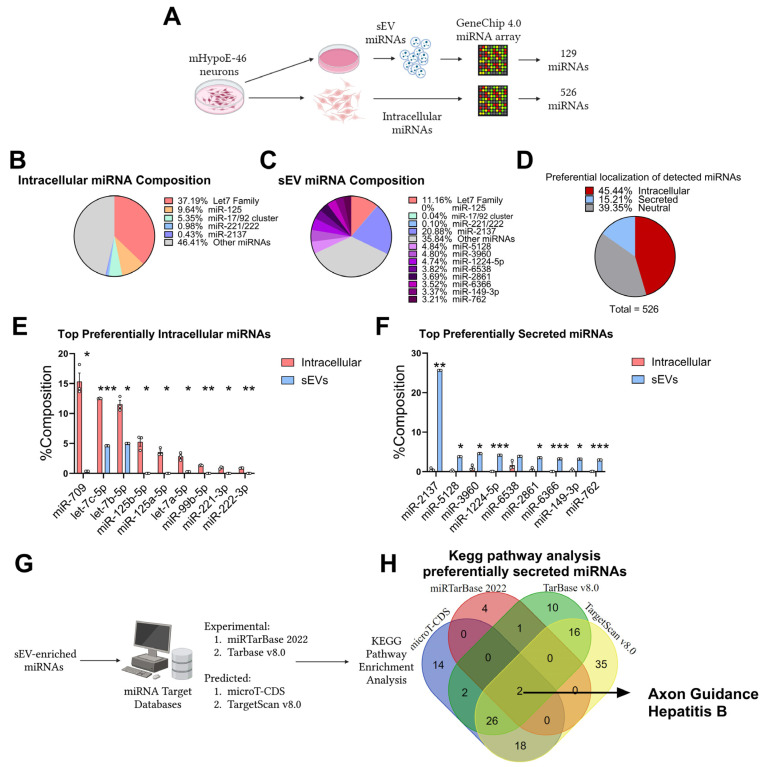
Specific miRNAs are preferentially secreted or retained in mHypoE-46 neurons. (**A**) Illustration depicting the assessment of miRNAs within intracellular compartments or small extracellular vesicles (sEVs) from mHypoE-46 neurons using the GeneChip 4.0 miRNA array. (**B**–**D**) Distribution of identified miRNAs categorized by their preferential secretion, cellular retention, or unbiased distribution. (**E**,**F**) Percentage composition of the top preferential intracellular or sEV miRNAs. (**G**) Diagram explaining the methodology for KEGG Pathway analysis of sEV-enriched miRNAs using multiple databases. (**H**) Venn diagram depicting pathway overlaps identified through independent KEGG pathway analyses across the four databases. Data are expressed as mean ± SEM. Statistics were determined using unpaired student *t*-test (E, F), adjusting for multiple comparisons using a two-stage setup method by Benjamini, Krieger, and Yekutieli. * *q* < 0.05, ** *q* < 0.01, *** *q* < 0.001. n = 3; independent cell culture preparations.

To compare the composition of intracellular and extracellular miRNA pools, an existing dataset previously published by our laboratory was assessed [18]. In mHypoE-46 neurons, the Genechip 4.0 microarray confidently detected (DABG, *p* < 0.05) 527 miRNAs. In the sEVs produced by these neurons, 129 miRNAs were confidently detected (Figure 1A). The percentage composition of each miRNA in each fraction was then compiled as described (Figure 1B,C). The composition of the two groups is significantly different. For example, the let-7 family constitutes 37.19% of the intracellular miRNAs but only 11.16% of the sEV miRNAs. Additionally, miR-2137, which accounts for about 0.43% of the intracellular miRNAs, comprises 20.88% of the sEV miRNAs. Out of the 526 detected miRNAs, 80 miRNAs were significantly enriched in sEV (15.21%) and 239 were significantly enriched intracellularly (45.44%), while 207 were neutral (39.35%) (Figure 1D). The top 10 miRNAs that were significantly enriched intracellularly were miR-709, let-7c-5p, let-7b-5p, miR-125b-5p, miR-125a-5p, and let-7a-5p, miR-99b-5p, miR-221-3p, and miR-222-3p. The top 10 miRNAs that were significantly enriched in sEVs were miR-2137, miR-5128, miR-3960, miR-1224-5p, miR-6538, miR-2861, miR-6366, miR-149-3p, and miR-762. These results demonstrate that miRNA secretion and cellular retention is actively regulated in mHypoE-46 neurons. Given that specific miRNAs are enriched in these neuronal sEVs, it is hypothesized that these miRNAs may be involved in intercellular communication. Four independent KEGG pathway analyses were conducted using targets from each of the four databases: miRTarBase 2022 (experimental), Tarbase v8.0 (experimental), microT-CDS 2023 (predicted), and TargetScan v8.0 (predicted) (Figure 1G). Axon guidance was significantly enriched in all four analyses, and this further supports the hypothesis that there is a functional enrichment of specific neuronal miRNAs in sEVs (Figure 1H, Supplementary Table S1).

### 3.2. RBP Motifs Statistically Correlate with the Differential Sorting of Neuronal miRNAs

**Figure 2:**
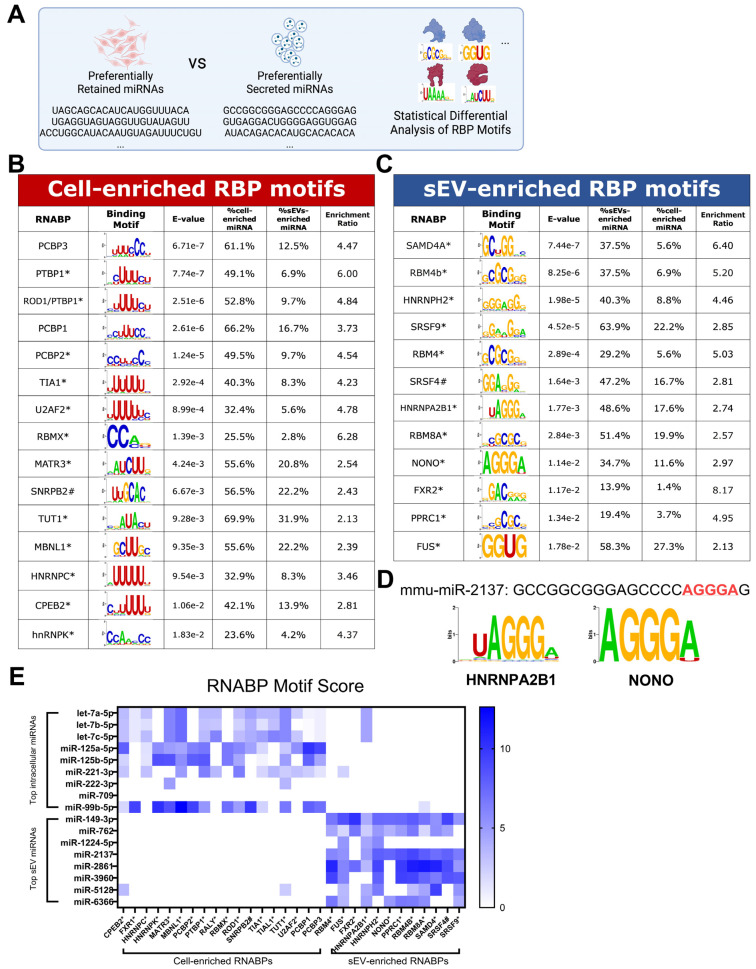
RNA-Binding Proteins’ Motifs Statistically Correlate with the Differential Sorting of Neuronal miRNAs. (**A**) Diagram illustrating the statistical enrichment analysis of recognized RBP motifs sourced from the CISBP-RNA database, assessing the significant overrepresentation of specific sequences. (**B**) RBPs statistically enriched within intracellular miRNAs. (**C**) RBPs statistically enriched within sEV miRNAs. (**D**) Illustration of mmu-miR-2137 sequence containing prominent HNRNPA2B1 and NONO motifs. (**E**) Heatmap depicting the specific clustering of top 10 intracellular or sEV miRNAs based on RBP motif strength. Annotations indicate motifs identified from homologous RBPs in other species: * *Homo sapiens*, ^#^ *Drosophila melanogaster*.

Subsequent analyses aimed to assess whether sEV and intracellularly enriched miRNAs were significantly enriched in the motifs of RBPs. The MEME suite, in conjunction with the CISBP-RNA database, was used to determine the motif enrichment in the two groups, as illustrated in Figure 2A [26,27]. The intracellularly enriched miRNAs displayed a significant enrichment in the binding sites for PCBP3, PTBP1, PCBP1, PCBP2, TIA1, U2AF2, RBMX, MATR3, SNRPB2, TUT1, MBNL1, HNRNPC, CPEB2, hnRNPK, TIAL1, and RALY. Conversely, sEV-enriched miRNAs were significantly enriched in the binding sites for SAMD4, RBM4B, HNRNPH2, SRSF9, RBM4, SRSF4, HNRNPA2B1, RBM8A, NONO, FXR2, PPRC1, and FUS. Notably, the sEV-enriched motifs included HNRNPA2B1, a protein previously shown to facilitate miRNA sorting into small extracellular vesicles, which confirms the validity of the current categorization and statistical analysis [8]. mRNA expression of these RBPs was detectable in mHypoE-46 neurons, as detailed in Supplementary Table S1. An illustration of an RBP motif is provided in Figure 2D, showing miR-2137, with the sequence GCCGGCGGGAGCCCCAGGGAG, containing an “AGGGA” motif that correlates highly with HNRNPA2B1 (uAGGGA) and NONO (AGGGA). A heat map reveals that the top 10 intracellular and sEV-enriched miRNAs can be distinguished based on the enriched RBP motifs (Figure 2E), suggesting that the presence of RBP motifs may indicate whether an miRNA is preferentially secreted or retained within the cell. sEV-enriched miRNAs, on average, contained a significantly lower AU%. This difference in AU% composition is also reflected across multiple cell models [19]. The average length of the sEV-enriched miRNAs is no different from that of the intracellularly enriched miRNAs (Supplementary Figure S1).

### 3.3. The Presence and Strength of RBP Motifs Can Be Used to Accurately Predict miRNA Localization in mHypoE-46 Neurons

**Figure 3:**
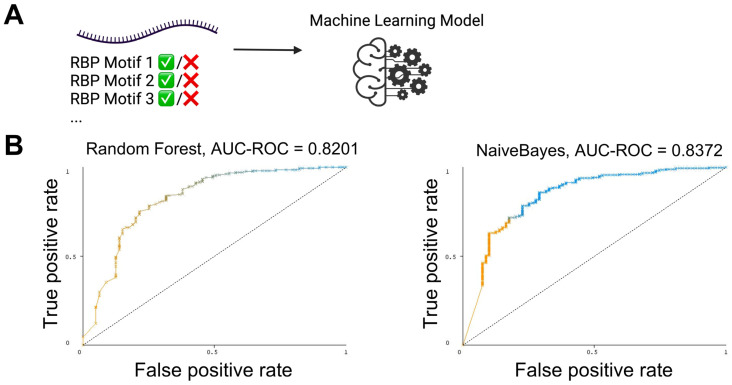
RBP Motifs Predict miRNA Localization in mHypoE-46 Neurons. (**A**) Architectural representation of machine learning methodologies leveraging the quantified strengths of identified RBP motifs to infer the preferential secretion or intracellular retention of miRNAs. The predictive model validation employs 10-fold cross-validation, supplemented by algorithmic analysis using Naïve Bayes and Random Forest classifiers. (**B**) Receiver operating characteristic (ROC) curves for Random Forest and Naïve Bayes Models.

If the identified RBPs and their corresponding motifs are indeed involved in the preferential localization of miRNAs, their combined presence and strength should offer a predictive power to determining whether an miRNA will be preferentially secreted or retained intracellularly. To test this hypothesis, the Weka 3.8.6 package was employed for machine learning modeling [28]. The presence and strength of the RBP motifs for each miRNA were used as features for training, utilizing either Random Forest or Naive Bayes, followed by 10-fold cross-validation (Figure 3A). In the mHypoE-46 neurons, the Random Forest model achieved an area under the receiver operating characteristic curve (AUC-ROC) of 0.8201, whereas Naive Bayes reached an AUC-ROC of 0.8372 (Figure 3B, Supplementary Table S2). A perfect AUC-ROC is 1.0. These models did not experience extensive optimization, and this range is deemed excellent, indicating the good discriminative ability of the models. Such levels of AUC are often satisfactory for practical applications, demonstrating that the model has a high rate of true positive predictions while maintaining a low rate of false positives. The accuracy of these models suggests that RBPs play a significant role in the sorting of microRNAs in mHypoE-46 neurons.

### 3.4. Enrichment of RBP Motifs in Preferentially Sorted miRNAs across Multiple Cell Types

**Figure 4:**
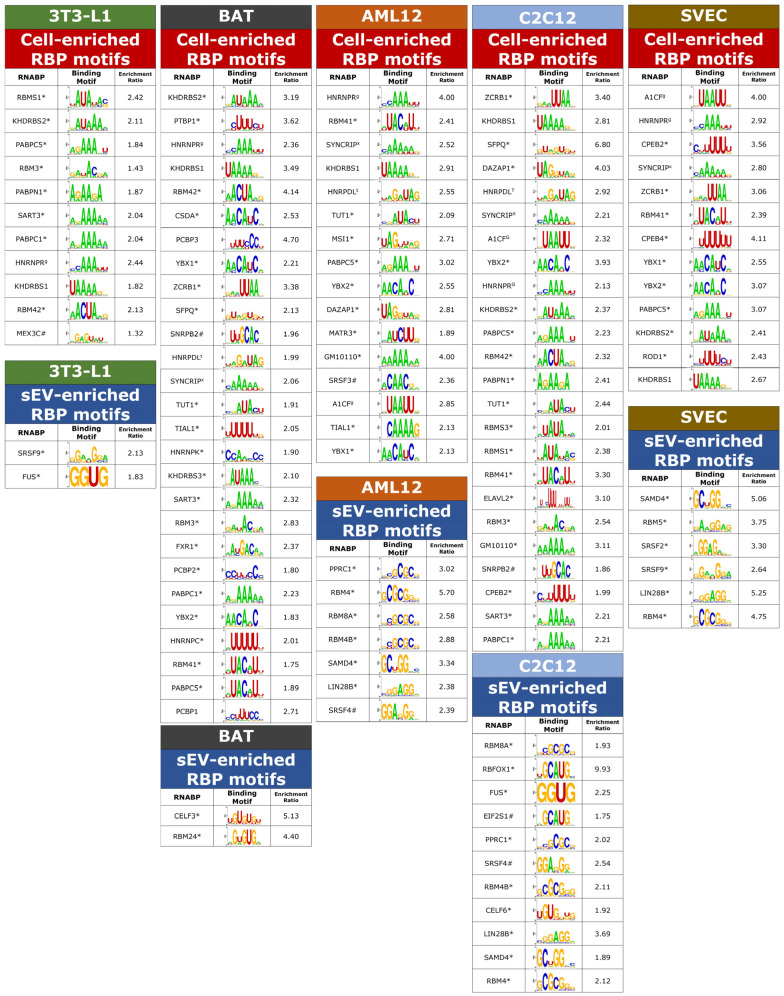
RBP motifs predict miRNA localization in multiple cell models. RBP motifs statistically enriched in sEVs or cells. Annotations indicate motifs identified from homologous RBPs in other species: * *Homo sapiens*, ^g^ *Gallus gallus*, ^t^ *Tetraodon nigroviridis*, ^x^ *Xenopus tropicalis*, ^#^ *Drosophila melanogaster*. The full extended figure can be found in the Supplementary Materials.

To investigate the role of RBPs in miRNA sorting across other mammalian cell types, we used published miRNA datasets from Garcia et al. [19]. The dataset includes pre-classified sEV and intracellular miRNA profiles from five distinct models: differentiated 3T3-L1 cells (white adipocytes), immortalized differentiated brown adipocytes, differentiated C2C12 cells (skeletal muscle), SVEC cells (endothelial cells), and AML12 cells (hepatocytes). These profiles represented independent quantification, statistical analysis, and classification into sEV/intracellular enriched miRNAs. Upon processing these datasets through the same analytical pipeline, multiple RBP motifs were identified as being differentially enriched in miRNAs, depending on the cell types (Figure 4). In 3T3-L1 cells, the motif for FUS (GGUG) was significantly enriched by 1.8-fold in sEV-enriched miRNAs. Garcia et al. also identified GGUG as a motif that is significantly enriched in sEV miRNAs in 3T3-L1 neurons, exhibiting the same fold change, which further supports the validity of the current analysis. Notably, certain RBP motifs were recurrent across the six different models. For example, sEV-enriched miRNAs that were present in at least four cell lines displayed enrichment for RBP motifs associated with RBM4 and SAMD4. In parallel, in all five non-neuronal cell lines, intracellular-enriched miRNAs exhibited enrichment of RBP motifs linked to HNRNPR, PABPC5, and KHDRBS1. Additionally, motifs for KHDRBS2, TUT1, YBX2, RBM41, A1CF, and SYNCRIP were cellularly enriched in at least four distinct cell lines. These findings from the datasets created by Garcia et al. reinforce their conclusion that the sequences of miRNAs play a crucial role in their sorting. Indeed, this sequence-based sorting may be predominantly mediated by RBPs.

### 3.5. The Presence and Strength of RBP Motifs Can Be Used to Accurately Predict miRNA Localization in Multiple Peripheral Models

**Figure 5:**
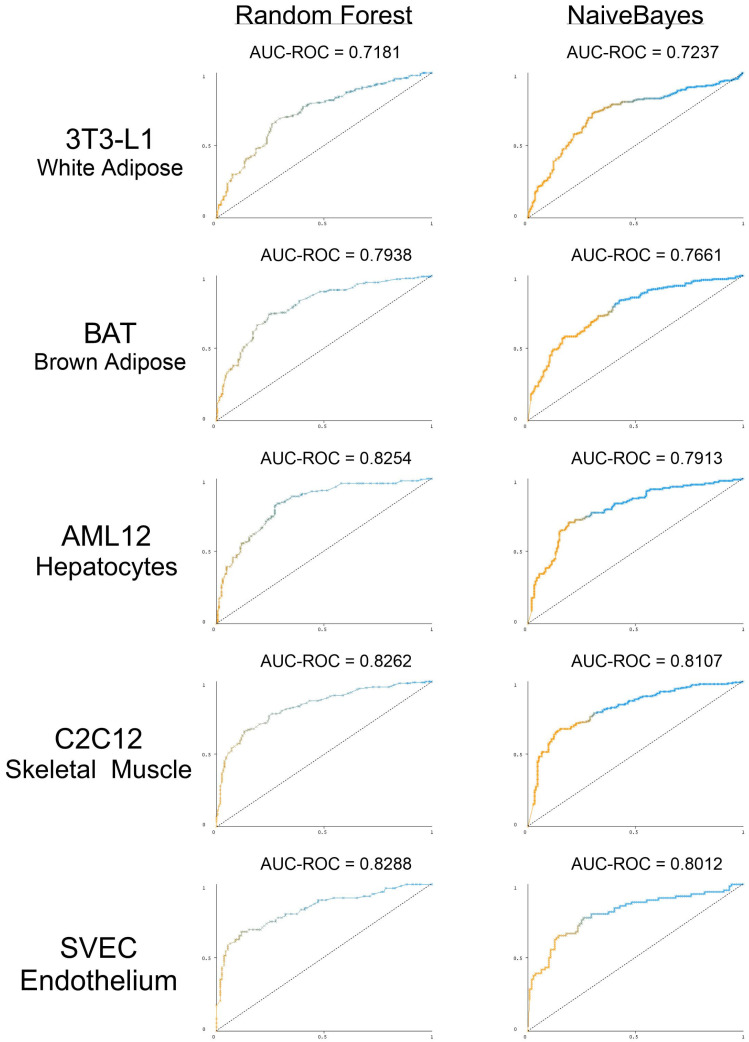
RBP motifs can be used to predict preferential miRNA localization in multiple mammalian cell models. Receiver operating characteristic (ROC) curves for Random Forest and Naïve Bayes Models for differentiated 3T3-L1 cells (white adipocytes), immortalized differentiated brown adipocytes, differentiated C2C12 cells (skeletal muscle), SVEC (endothelial cells), and AML12 cells (hepatocytes). The Y-axis represents the true positive rate, while the X-axis represents the false positive rate.

While multiple motifs have been identified in each of the peripheral cell models, it is still unclear whether these motifs serve any predictive power in terms of miRNA distribution. The same machine learning pipeline was applied to determine if cell-line-specific RBP motifs could predict cell-line-specific miRNA secretion and retention. In 3T3 white adipocytes, the Random Forest model achieved an AUC-ROC score of 0.7181, slightly lower than the score for the Naive Bayes model of 0.7237. Superior performances were observed for other models, similar to those noted for mHypoE-46 neurons. In brown adipose tissue (BAT), the scores were 0.7938 for the Random Forest model and 0.7661 for Naive Bayes. AML12 cells saw the Random Forest model reaching an AUC-ROC of 0.8254, with the Naive Bayes model reached 0.7913. For C2C12 cells, the scores were 0.8262 for Random Forest and 0.8107 for Naive Bayes. In SVEC cells, the scores were 0.8288 for Random Forest and 0.8012 for Naive Bayes (Figure 5 and Supplementary Table S3). With the exception of the white adipocytes, the performance across the remaining models underscores the significant enhancement in predictive accuracy attributed to the presence and strength of RBP motifs. These results collectively underscore the critical role that RBPs play in miRNA sorting across unique mammalian cell types.

## 4. Discussion

This study provides significant bioinformatic evidence that multiple RBPs play a significant role in the localization of miRNAs in mammalian cells. Specifically, this study demonstrates the presence of regulated miRNA secretion and cellular retention in an NPY/AgRP hypothalamic neuronal model. Pathway analysis demonstrates that sEV-enriched miRNAs are involved in axon guidance, which suggests that functional intercellular communication is carried out by preferentially secreted miRNAs. Known RBP motifs are significantly enriched in differentially localized miRNAs and can be used to predict preferential miRNA secretion with a high level of accuracy. Relevance of RBP in miRNA sorting was further tested using publicly accessible sEV and intracellular miRNA profiles published by Garcia et al., 2021. Similar to the mHypoE-46 neurons, multiple RBP motifs are differentially enriched in the two fractions, and these motifs can be used to accurately predict cell-type specific miRNA secretion or cellular retention (AUC-ROC around 0.8). Selectively expressed miRNAs promote intercellular communication by being preferentially secreted due to the presence of specific RBPs and, upon reaching host cells, may encounter an environment lacking these RBPs or presenting a different subset, affecting their retention for gene regulation. Understanding the complete miRNA sorting machinery will enhance our comprehension of the origins of circulating miRNAs and their contribution to inter-cellular, -tissue and -organ communication.

The miRNA distribution patterns were not the same across the cell lines; likewise, the RBP motifs were not identical across cell lines. The RBM4 and SAMD4 motifs were significantly enriched in sEV-enriched miRNAs in four out of the six models presented. RBM4 (RNA Binding Motif Protein 4) is an RBP that regulates mRNA splicing, and RBM4 physically interacts with Argonaute 2 (AGO2) and recruits AGO2 to suppress the translation of target mRNAs [29,30]. SAMD4 is also linked to the Argonaute 1 (AGO1) protein, as its drosophila homolog Smaug directly recruits AGO1 to target mRNAs [31]. Sart3, whose motif is enriched in intracellular miRNAs from 3T3-L1, BAT, and C2C12, physically associates with the human AGO protein [29]. RBPs like SAMD4 and RBM4 may have a higher affinity for certain Ago-miRNA complexes through binding to defined miRNAs, thereby facilitating preferential export. Further experimental validation is necessary to ascertain the specific roles of these RBPs in miRNA sorting. For example, the evidence suggests that LC3-dependent, autophagy-mediated packaging could play a role in the secretion of select RBPs [32].

Out of all the models, white adipocytes (3T3-L1) produced the worst performing machine learning model (AUC-ROC around 0.7), which indicates that the RBPs may play a lesser role in regulating miRNA secretion and cellular retention in this model. In support of this, comparing the percentage composition of miRNAs based off the RNA-seq data, similarly to the method used for the mHypoE-46 neurons presented in this study, resulted in there being no significant difference in the miRNA composition in terms of sEV content and intracellular content (data taken from Garcia et al.) [19]. However, AML12, which showed a significantly better AUC-ROC (0.8+), undergoing this analysis still resulted in differentially enriched miRNAs. These observations suggest that the 3T3-L1 cell line, a model of adipocytes that undergo a defined differentiation protocol, and by extension, potentially white adipose tissue, has a lesser discriminative secretion and retention of miRNAs. White adipose tissue is unique compared to other tissues because it contributes to most of the circulating miRNAs [33]. Indeed, 3T3-L1 can secrete up to 800-fold more sEV per cell compared to other cell types, which either indicates an anomaly within this cell line itself or an extremely active sEV secretory process [19]. The regulation of in vivo WAT sEV secretion, and by extension miRNA signaling, may be regulated by other mechanisms, such as the tissue-specific targeting of the sEV themselves.

The current machine learning models used exhibited promising results, yet there is potential for further improvement, as significant optimization was not explored in this study. For instance, due to the high correlation among certain motifs, it is conceivable that a smaller set of RBP motifs could retain the same predictive power. In parallel, not all potentially relevant RBPs were included in the study given the fact that many RBPs do not have experimentally defined motifs. Modelling with all motifs, not just those that are significantly enriched, might prove beneficial. The weighting of different RBP motifs may be adjusted by incorporating mRNA or protein expression data for the RBP. Limitations should also be addressed in the future. Most of the RBP motifs used in this study are based on highly homologous RBP proteins, which may exhibit slight variations in their motifs compared to the mouse RBPs pertinent to this research. Binary classification was used in the study, excluding the neutral group because is most likely contains significant misclassifications due to current limitations in miRNA quantification [20]. While RBPs alone demonstrate significant predictive capabilities, it is possible that other factors also play a role in the systematic sorting of miRNAs. Addressing these factors in the models could lead to more accurate predictions. Such factors include the intracellular and sEV levels of the mRNA/long non-coding RNA (lncRNA) targets of an miRNA [34], highlighting a broader context for a potential improvement in predictive accuracy.

The robustness of current machine learning models highlights their potential for further refinement in the development of small RNA therapeutics. Two small interfering RNAs (Patisiran/ALN-TTR02 and Givosiran/ALN-AS1) are approved for use to treat rare genetic conditions [35]. Toxic antisense oligonucleotides are associated with multiple RBPs [3], so engineering therapeutics with RBP motifs in mind may prove beneficial to reduce their off-target effects. Given that miRNA mimics and therapeutics often employ chemical modifications to the backbone or nitrogenous base, future investigations are needed to assess how these modifications influence RBP-miRNA interactions. Further, the addition of a cellular retention motif may enhance the delivery and retention of exogenous miRNA mimics or inhibitors [20]. For instance, the delivered miRNA may be retained in the cell longer with such a motif, while a targeted small-RNA drug design may be used in combination with other cellular and biochemical manipulations to modulate the pharmacokinetics of the delivered miRNAs. Thus, a greater understanding of RBP-RNA interactions is key to maximizing delivery potential and minimizing off-target effects.

In conclusion, the current study demonstrates regulated miRNA secretion in NPY/AgRP hypothalamic neurons. This regulated secretion can be attributed to the sequence of miRNAs. Specifically, the presence and strength of RBP motifs predicts whether an miRNA would be preferentially secreted or retained in the cell. The same conclusion extends to non-neuronal models, including white adipose tissue, brown adipose tissue, skeletal muscle, endothelial cells, and hepatocytes. Overall, this study provides significant evidence that RBPs play a prominent role in miRNA localization. Understanding the regulated secretion of miRNAs is essential for unraveling complex intercellular and intratissue communications, while also offering potential therapeutic applications for small RNAs.

## Data Availability

Relevant information can be found in the Supplementary Materials. The data presented in this study are available on request from the corresponding author.

## Supplementary Materials

The following supporting information can be downloaded at: https://www.mdpi.com/article/10.3390/biomedicines12040857/s1, Figure S1: AU% and miRNA length of sEV or intracellularly enriched miRNAs; Table S1: Complete pathway analysis; Table S2: RNA-seq levels of the RBPs in mHypoE-46 neurons; Table S3: Detailed Machine Learning Model Statistics.
