# Supplementary material for: RNA-Binding Protein Motifs Predict microRNA Secretion and Cellular Retention in Hypothalamic and Other Cell Types
Source: Biomedicines. 2024 Apr 12;12(4):857. doi: 10.3390/biomedicines12040857 (PMC11048351; doi:10.3390/biomedicines12040857)
Supplement: Supplementary file 1 [file biomedicines-12-00857-s001.zip › biomedicines-2928525-supplementary.pdf]

Supplemental Materials

RNA binding protein motifs predict microRNA secretion and cellular retention in hypothalamic and other cell types.

Wenyuan He[1] and Denise D. Belsham[1,2] *

[1]Departments of Physiology, University of Toronto, Ontario, Canada.

[2] Departments of Medicine, University of Toronto, Ontario, Canada.

* Corresponding Author at

Department of Physiology, University of Toronto, Medical Sciences Building 3247A, 1 Kings College Circle, Toronto, ON, Canada, M5S 1A8

Tel.: 416-946-7646; Fax: 416-978-4940; E-mail address: d.belsham@utoronto.ca

Extended Figure 4

RBP motifs statistically enriched in sEVs or cells. Annotations indicate motifs identified from homologous RNA-binding proteins (RNABPs) in other species: **Homo sapiens*, [g]*Gallus gallus*, [t]*Tetraodon nigroviridis*, [x]*Xenopus tropicalis*, #*Drosophila melanogaster*.

3T3-L1 — Cell-enriched RBP motifs

RNABP	Binding Motif	E-value	%cell-enriched miRNA	%sEVs-enriched miRNA	Enrichment Ratio
RBMS1*		1.04e-3	35.7%	14.4%	2.42
KHDRBS2*		1.34e-3	42.0%	19.6%	2.11
PABPC5*		1.60e-3	51.0%	27.5%	1.84
RBM3*		1.86e-3	75.8%	52.9%	1.43
PABPN1*		3.05e-3	47.1%	24.8%	1.87
SART3*		3.50e-3	40.8%	19.6%	2.04
PABPC1*		3.50e-3	40.8%	19.6%	2.04
HNRNPR[g]		4.31e-3	31.2%	12.4%	2.44
KHDRBS1		1.09e-2	44.6%	24.2%	1.82
RBM42*		3.88e-2	29.9%	13.7%	2.13
MEX3C#		4.79e-2	74.5%	56.2%	1.32

3T3-L1 — sEV-enriched RBP motifs

RNABP	Binding Motif	E-value	%sEVs-enriched miRNA	%cell-enriched miRNA	Enrichment Ratio
SRSF9*		2.49e-3	54.9%	25.5%	2.13
FUS*		4.85e-2	52.9%	28.7%	1.83

BAT — Cell-enriched RBP motifs

RNABP	Binding Motif	E-value	%cell-enriched miRNA	%sEVs-enriched miRNA	Enrichment Ratio
KHDRBS2*		4.42e-6	46.5%	14.1%	3.19
PTBP1*		9.33e-5	31.9%	8.3%	3.62
HNRNPR[g]		9.87e-5	58.4%	24.4%	2.36
KHDRBS1		1.01e-4	33.0%	9.0%	3.49
RBM42*		2.07e-4	25.9%	5.8%	4.14
CSDA*		3.11e-4	46.5%	17.9%	2.53
PCBP3		7.16e-4	20.5%	3.8%	4.70
YBX1*		8.25e-4	54.6%	24.4%	2.21
ZCRB1*		1.02e-3	27.6%	7.7%	3.38
SFPQ*		1.83e-3	54.1%	25.0%	2.13
SNRPB2#		1.86e-3	65.9%	33.3%	1.96
HNRPDL[t]		3.08e-3	59.5%	29.5%	1.99
SYNCRIP[x]		3.92e-3	53.5%	25.6%	2.06
TUT1*		3.92e-3	64.3%	33.3%	1.91
TIAL1*		9.52e-3	48.1%	23.1%	2.05
HNRNPK*		1.13e-2	56.8%	29.5%	1.90
KHDRBS3*		1.35e-2	43.8%	20.5%	2.10
SART3*		1.42e-2	35.1%	14.7%	2.32
RBM3*		1.54e-2	24.9%	8.3%	2.83
FXR1*		2.37e-2	31.4%	12.8%	2.37
PCBP2*		2.39e-2	59.5%	32.7%	1.80
PABPC1*		2.46e-2	35.1%	15.4%	2.23
YBX2*		2.51e-2	56.8%	30.8%	1.83
HNRNPC*		2.81e-2	43.2%	21.2%	2.01
RBM41*		2.94e-2	63.2%	35.9%	1.75
PABPC5*		2.97e-2	50.3%	26.3%	1.89
PCBP1		3.22e-2	23.8%	8.3%	2.71
MATR3*		3.66e-2	56.8%	31.4%	1.79
TIA1*		3.68e-2	42.7%	21.2%	1.99
A1CF[g]		3.94e-2	47.0%	24.4%	1.90
DAZAP1*		4.90e-2	49.2%	26.3%	1.85

BAT — sEV-enriched RBP motifs

RNABP	Binding Motif	E-value	%sEVs-enriched miRNA	%cell-enriched miRNA	Enrichment Ratio
CELF3*		2.39e-3	2.7%	16.0%	5.13
RBM24*		6.94e-3	3.2%	16.0%	4.40

AML12 — Cell-enriched RBP motifs

RNABP	Binding Motif	E-value	%cell-enriched miRNA	%sEVs-enriched miRNA	Enrichment Ratio
HNRNPR[g]		3.47e-6	49.7%	11.7%	4.00
RBM41*		1.10e-4	75.2%	30.6%	2.41
SYNCRIP[x]		7.49e-4	58.2%	22.5%	2.52
KHDRBS1		7.52e-4	46.4%	15.3%	2.91
HNRPDL[t]		1.22e-3	54.2%	20.7%	2.55
TUT1*		3.77e-3	70.6%	33.3%	2.09
MSI1*		3.80e-3	43.1%	15.3%	2.71
PABPC5*		6.12e-3	34.6%	10.8%	3.02
YBX2*		1.32e-2	40.5%	15.3%	2.55
DAZAP1*		1.34e-2	34.6%	11.7%	2.81
MATR3*		1.43e-2	75.8%	39.6%	1.89
Gm10110*		2.15e-2	20.9%	4.5%	4.00
SRSF3#		2.61e-2	41.8%	17.1%	2.36
A1CF[g]		2.96e-2	30.1%	9.9%	2.85
TIAL1*		3.78e-2	49.0%	22.5%	2.13
YBX1*		3.78e-2	49.0%	22.5%	2.13

AML12 — sEV-enriched RBP motifs

RNABP	Binding Motif	E-value	%sEVs-enriched miRNA	%cell-enriched miRNA	Enrichment Ratio
Pprc1*		1.06e-4	48.6%	15.7%	3.02
RBM4*		1.62e-4	25.2%	3.9%	5.70
Rbm8a*		3.72e-4	55.0%	20.9%	2.58
Rbm4b*		1.51e-3	40.5%	13.7%	2.88
Samd4*		4.12e-3	29.7%	8.5%	3.34
Lin28b*		2.10e-2	39.6%	16.3%	2.38
Srsf4#		4.34e-2	35.1%	14.4%	2.39

C2C12 — Cell-enriched RBP motifs

RNABP	Binding Motif	E-value	%cell-enriched miRNA	%sEVs-enriched miRNA	Enrichment Ratio
ZCRB1*		1.01e-6	57.1%	16.3%	3.40
KHDRBS1		6.20e-5	55.0%	19.1%	2.81
Sfpq*		8.19e-4	18.6%	2.1%	6.80
Dazap1*		8.56e-4	27.9%	6.4%	4.03
HNRPDL[t]		8.79e-4	40.7%	13.5%	2.92
SYNCRIP[x]		1.03e-3	65.0%	29.1%	2.21
A1CF[g]		1.04e-3	58.6%	24.8%	2.32
YBX2*		1.39e-3	27.1%	6.4%	3.93
HNRNPR[g]		1.60e-3	67.1%	31.2%	2.13
Khdrbs2*		2.36e-3	51.4%	21.3%	2.37
Pabpc5*		2.40e-3	57.9%	25.5%	2.23
Rbm42*		2.40e-3	53.6%	22.7%	2.32
Pabpn1*		3.86e-3	47.1%	19.1%	2.41
Tut1*		5.34e-3	44.3%	17.7%	2.44
Rbms3*		7.37e-3	63.6%	31.2%	2.01
Rbms1*		1.29e-2	41.4%	17.0%	2.38
Rbm41*		1.35e-2	25.0%	7.1%	3.30
Elavl2*		1.35e-2	27.9%	8.5%	3.10
Rbm3*		2.37e-2	33.6%	12.8%	2.54
Gm10110*		3.27e-2	23.6%	7.1%	3.11
Snrpb2#		3.91e-2	61.4%	32.6%	1.86
Cpeb2*		4.02e-2	51.4%	25.5%	1.99
Sart3*		4.28e-2	40.0%	17.7%	2.21
Pabpc1*		4.28e-2	40.0%	17.7%	2.21

C2C12 — sEV-enriched RBP motifs

RNABP	Binding Motif	E-value	%sEVs-enriched miRNA	%cell-enriched miRNA	Enrichment Ratio
RBM8A*		6.54e-6	53.2%	27.1%	1.93
RBFOX1*		1.19e-5	13.5%	0.7%	9.93
FUS*		1.43e-5	41.1%	17.9%	2.25
EIF2S1#		1.60e-5	58.2%	32.9%	1.75
PPRC1*		6.33e-5	42.6%	20.7%	2.02
SRSF4#		1.44e-4	28.4%	10.7%	2.54
RBM4B*		1.48e-4	36.9%	17.1%	2.11
CELF6*		1.78e-4	41.8%	21.4%	1.92
LIN28B*		2.35e-4	17.7%	4.3%	3.69
SAMD4*		3.95e-4	39.7%	20.7%	1.89
RBM4*		3.99e-4	32.6%	15.0%	2.12

SVEC — Cell-enriched RBP motifs

RNABP	Binding Motif	E-value	%cell-enriched miRNA	%sEVs-enriched miRNA	Enrichment Ratio
A1CF[g]		1.78e-4	58.4%	13.4%	4.00
HNRNPR[g]		1.52e-3	68.3%	22.4%	2.92
CPEB2*		4.80e-3	46.5%	11.9%	3.56
SYNCRIP[x]		6.12e-3	61.4%	20.9%	2.80
ZCRB1*		6.98e-3	53.5%	16.4%	3.06
RBM41*		7.67e-3	77.2%	31.3%	2.39
CPEB4*		1.14e-2	35.6%	7.5%	4.11
YBX1*		1.45e-2	63.4%	23.9%	2.55
YBX2*		2.40e-2	44.6%	13.4%	3.07
PABPC5*		2.40e-2	44.6%	13.4%	3.07
KHDRBS2*		2.82e-2	63.4%	25.4%	2.41
ROD1*		3.41e-2	60.4%	23.9%	2.43
KHDRBS1		3.91e-2	50.5%	17.9%	2.67

SVEC — sEV-enriched RBP motifs

RNABP	Binding Motif	E-value	%sEVs-enriched miRNA	%cell-enriched miRNA	Enrichment Ratio
SAMD4*		5.66e-4	38.8%	6.9%	5.06
RBM5*		2.76e-3	43.3%	10.9%	3.75
SRSF2*		4.15e-3	47.8%	13.9%	3.30
SRSF9*		4.73e-3	64.2%	23.8%	2.64
LIN28B*		4.75e-3	29.9%	5.0%	5.25
RBM4*		1.93e-2	26.9%	5.0%	4.75

Supplemental Figure S1: AU% content and miRNA length of sEV or intracellularly enriched miRNAs. The percentage of AU versus GC composition was examined in sEV-enriched miRNAs versus miRNAs enriched intracellularly in mHypoE-46 neurons. Consistent with observations made by Garcia et al., 2021, sEV-enriched miRNAs, on average, contained a significantly lower AU%. This difference in AU% composition across multiple cell models is also reflected in the enriched RBP motifs, where sEV-enriched miRNAs typically have RBP motifs that are more CG-enriched than those enriched intracellularly. The average length of the sEV-enriched miRNAs is no different from that of the intracellularly enriched miRNAs.

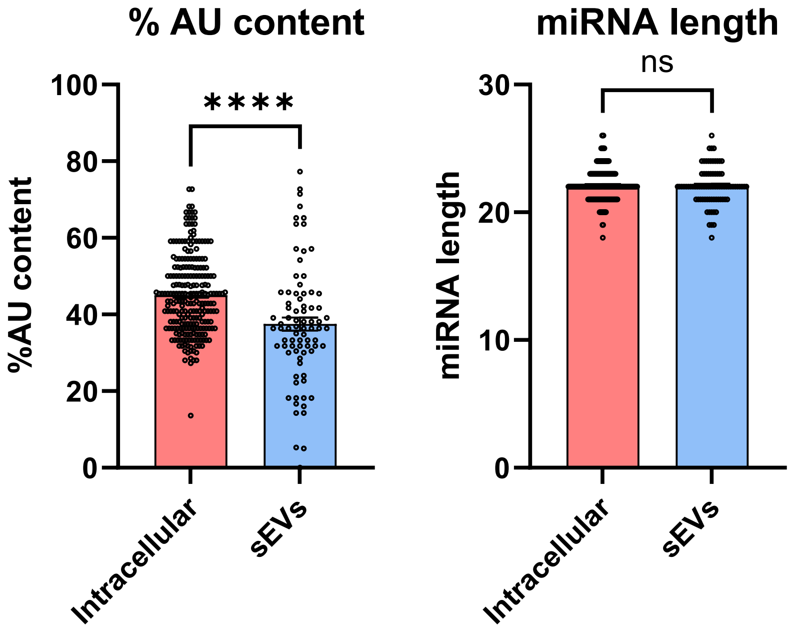

Supplemental Table S1: Complete pathway analysis.

See excel document – He and Belsham Supplemental Table S1.

Supplemental Table S2: RNA-seq levels of the RBPs in mHypoE-46 neurons. Comparative mRNA expression levels of identified RBPs in hypothalamic neurons as determined by RNA-sequencing, with Rpl7 serving as a reference gene. Number represents fragments per kilobase of transcript per million mapped reads.

Pcbp3	7.3		Samd4	14.4
Ptbp1	243.5		Rbm4b	2
Pcbp1	257.4		Hnrnph2	52.9
Pcbp2	263.1		Srsf9	84.6
Tia1	11.9		Rbm4	8.7
U2af2	155.5		Srsf4	35.1
Rbmx	13.6		Hnrnpa2b1	434.6
Matr3	48.2		Rbm8a	55.2
Snrpb2	47.1		Nono	229.3
Tut1	21.8		Fxr2	55.4
Mbnl1	45.1		Pprc1	40.4
Hnrnpc	152.5		Fus	127.3
Cpeb2	2.1			
Hnrnpk	273.3			
Tial1	18.2			
Ralyl	0		Rpl7	530.9

Supplemental Table S3: Detailed machine learning model statistics.

Model/Class	TP	FP	Precision	Recall	F-Measure	MCC	ROC Area	PRC Area
**N46 - RF**								
Intracellular	0.929	0.456	0.86	0.929	0.893	0.523	0.82	0.908
sEV	0.544	0.071	0.717	0.544	0.619	0.523	0.82	0.678
Weighted	0.833	0.36	0.825	0.833	0.825	0.523	0.82	0.851
**N46 - NB**								
Intracellular	0.703	0.177	0.923	0.703	0.798	0.459	0.837	0.918
sEV	0.823	0.297	0.478	0.823	0.605	0.459	0.835	0.669
Weighted	0.733	0.207	0.812	0.733	0.75	0.459	0.837	0.856
**3T3-L1 - RF**								
Intracellular	0.724	0.341	0.685	0.724	0.704	0.384	0.724	0.713
sEV	0.659	0.276	0.7	0.659	0.679	0.384	0.724	0.702
Weighted	0.692	0.309	0.692	0.692	0.691	0.384	0.724	0.708
**3T3-L1 - NB**								
Intracellular	0.546	0.2	0.736	0.546	0.627	0.357	0.718	0.661
sEV	0.8	0.454	0.633	0.8	0.706	0.357	0.718	0.702
Weighted	0.672	0.326	0.685	0.672	0.666	0.357	0.718	0.681
**BAT - RF**								
Intracellular	0.78	0.329	0.737	0.78	0.758	0.454	0.794	0.795
sEV	0.671	0.22	0.72	0.671	0.695	0.454	0.794	0.757
Weighted	0.73	0.279	0.73	0.73	0.729	0.454	0.794	0.778
**BAT - NB**								
Intracellular	0.605	0.231	0.756	0.605	0.672	0.376	0.766	0.778
sEV	0.769	0.395	0.621	0.769	0.687	0.376	0.766	0.731
Weighted	0.68	0.306	0.694	0.68	0.679	0.376	0.766	0.757
**AML12 - RF**								
Intracellular	0.822	0.382	0.747	0.822	0.783	0.452	0.825	0.852
sEV	0.618	0.178	0.717	0.618	0.664	0.452	0.825	0.754
Weighted	0.736	0.296	0.735	0.736	0.733	0.452	0.825	0.811
**AML12 - NB**								
Intracellular	0.734	0.268	0.79	0.734	0.761	0.461	0.792	0.816
sEV	0.732	0.266	0.667	0.732	0.698	0.461	0.791	0.719
Weighted	0.733	0.267	0.738	0.733	0.734	0.461	0.792	0.775
**C2C12 - RF**								
Intracellular	0.8	0.295	0.729	0.8	0.763	0.507	0.826	0.799
sEV	0.705	0.2	0.78	0.705	0.741	0.507	0.826	0.832

Weighted	0.752	0.247	0.755	0.752	0.752	0.507	0.826	0.816
**C2C12 - NB**								
Intracellular	0.703	0.244	0.741	0.703	0.722	0.46	0.81	0.784
sEV	0.756	0.297	0.72	0.756	0.738	0.46	0.811	0.798
Weighted	0.73	0.27	0.73	0.73	0.73	0.46	0.811	0.791
**SVEC - RF**								
Intracellular	0.866	0.324	0.802	0.866	0.833	0.556	0.829	0.857
sEV	0.676	0.134	0.769	0.676	0.719	0.556	0.829	0.802
Weighted	0.79	0.249	0.789	0.79	0.788	0.556	0.829	0.835
**SVEC - NB**								
Intracellular	0.768	0.284	0.804	0.768	0.785	0.479	0.802	0.813
sEV	0.716	0.232	0.671	0.716	0.693	0.479	0.801	0.762
Weighted	0.747	0.263	0.751	0.747	0.749	0.479	0.802	0.793
